# Link between Epigenomic Alterations and Genome-Wide Aberrant Transcriptional Response to Allergen in Dendritic Cells Conveying Maternal Asthma Risk

**DOI:** 10.1371/journal.pone.0070387

**Published:** 2013-08-12

**Authors:** Lyudmila Mikhaylova, Yiming Zhang, Lester Kobzik, Alexey V. Fedulov

**Affiliations:** 1 Division of Pulmonary and Critical Care Medicine, Brigham and Women's Hospital, Harvard Medical School, Boston, Massachusetts, United States of America; 2 Department of Environmental Health, Harvard School of Public Health, Boston, Massachusetts, United States of America; University of North Dakota, United States of America

## Abstract

We investigated the link between epigenome-wide methylation aberrations at birth and genomic transcriptional changes upon allergen sensitization that occur in the neonatal dendritic cells (DC) due to maternal asthma. We previously demonstrated that neonates of asthmatic mothers are born with a functional skew in splenic DCs that can be seen even in allergen-naïve pups and can convey allergy responses to normal recipients. However, minimal-to-no transcriptional or phenotypic changes were found to explain this alteration. Here we provide in-depth analysis of genome-wide DNA methylation profiles and RNA transcriptional (microarray) profiles before and after allergen sensitization. We identified differentially methylated and differentially expressed loci and performed manually-curated matching of methylation status of the key regulatory sequences (promoters and CpG islands) to expression of their respective transcripts before and after sensitization. We found that while allergen-naive DCs from asthma-at-risk neonates have minimal transcriptional change compared to controls, the methylation changes are extensive. The substantial transcriptional change only becomes evident upon allergen sensitization, when it occurs in multiple genes with the pre-existing epigenetic alterations. We demonstrate that maternal asthma leads to both hyper- and hypomethylation in neonatal DCs, and that both types of events at various loci significantly overlap with transcriptional responses to allergen. Pathway analysis indicates that approximately 1/2 of differentially expressed and differentially methylated genes directly interact in known networks involved in allergy and asthma processes. We conclude that congenital epigenetic changes in DCs are strongly linked to altered transcriptional responses to allergen and to early-life asthma origin. The findings are consistent with the emerging paradigm that asthma is a disease with underlying epigenetic changes.

## Introduction

Allergy and, more specifically, allergic asthma, often starts early in life [1]–[6]. The onset of the disease is crucially linked to the decision-making point in the immune system, when the machinery of dendritic cells (DC) determines whether or not the protein is recognized as allergen for presentation to T-cells in a particular context [7]–[10]. This leads to development of a Th_2_ milieu that later maintains the allergy process [11]–[13]. The decision-making mechanism in the DCs is unknown; this hinders our understanding of how allergy originates. In our mouse model [14] genetically and environmentally identical neonates of asthmatic mothers develop allergy more readily compared to control counterparts coming from normal parents or asthmatic fathers. This model mirrors epidemiologic studies in humans [15]–[19] and indicates non-genetic and non-environmental transmission of asthma risk from the mother. The asthma-at-risk pups develop asthmatic symptoms in response to an intentionally suboptimal protocol (lower allergen dose/fewer administrations) in contrast to normal pups which do not develop symptoms under the same conditions. In adoptive transfer experiments [20] DCs from asthma-at-risk pups to normal pups, but not macrophages, CD4 T-cells, or DC-depleted splenocytes, caused increased susceptibility to asthma in the recipients, indicating that DCs of asthma-at-risk pups are skewed early in life to induce allergic responses.

In this study we have tested a hypothesis that maternal “risk inheritance” in our mouse model is conveyed via epigenetic changes occurring in pups prenatally or in early postnatal period. We chose splenic DCs as the object of our study because of their crucial role in adaptive immune responses, and because they are sufficient to cause an asthma-at-risk phenotype in the adoptive transfer experiments discussed above [20]. We assessed whole genome epigenetic and transcriptional changes in dendritic cells from normal and asthma-at-risk pups prior to (naïve pups) and after antigen sensitization (sensitized pups). The hypothesis was that maternal allergy induces epigenetic changes that can be detected at birth, and prior to allergen sensitization. We further postulated that, for these DNA methylation changes to lead to transcriptional difference in response to allergen, they would be found in key regulatory areas (e.g. promoters and CpG islands) of relevant genes. We therefore sought to detect and characterize DNA-wide methylation changes, and then to match them to differentially expressed transcripts. We further postulated that these altered methylation sites are likely to be linked causally to an early-life asthma risk phenotype. A final prediction tested was that ‘activation’ or ‘priming’ of the DC with allergen sensitization would reveal more transcriptional differences than would be found in unstimulated DCs from allergen-naive neonates.

## Results

### Asthma-at-risk DCs show large-scale DNA methylation differences compared to control

We found a large number of probes differentially methylated between naïve normal and asthma-at-risk pups. In contrast, only a small number of genes showed differential transcription under these conditions. However, a much greater number of differentially expressed genes was detected in DCs from allergen-stimulated pups. We found substantial overlap between genes differentially expressed in allergen-stimulated pups, and those containing differentially methylated probes in naïve pups. Pathway analysis of the overlapping genes revealed that a majority belong to several important transcriptional networks including those regulated by well-known inflammatory factors such as Nfkb and Stat1, as well as networks associated with cell differentiation and activation.

Specifically, we have analyzed a dataset (GSE13380) obtained using a Switchgear platform to identify differentially methylated regions (DMRs) in mouse dendritic cell DNA from normal mice and asthma-at-risk mice (offspring of normal and asthmatic mothers, respectively). The analysis revealed significant differences in methylation patterns between the two groups. Out of 345,225 experimental probes, we have found 23,477 probes (∼6.8%) differentially unmethylated in normal samples and 28,615 (∼8.3%) differentially unmethylated in asthma using a false discovery rate (FDR) of 0.05. 88,445 probes (∼26%) were found to be unmethylated in both groups. We followed by analyzing the results obtained from each group separately. We imposed additional restrictions to select the most relevant DMRs. First, we required that higher methylated samples for each DMR had log2 fold change >0.5 thus removing probes with ambiguous calls. Second, we have removed DMRs with absolute difference in log2 fold change between groups <0.3, to select the regions where the difference would more likely to be biologically relevant. This filtering resulted in 4,441 probes that we consider unmethylated in normal samples but methylated in asthma-at-risk, and 8,030 probes unmethylated in asthma-at-risk but methylated in normal samples. These DMRs were mapped respectively to 4,785 and 7,527 transcripts and/or promoters (defined as −1000 to +500 bp relative to the transcription start site), out of total 24,364 transcripts that had methylation probes mapping either within the transcript or in its promoter region.

### Transcriptional differences in naïve DCs are minor but correlate with epigenetic changes

**Figure 1:**
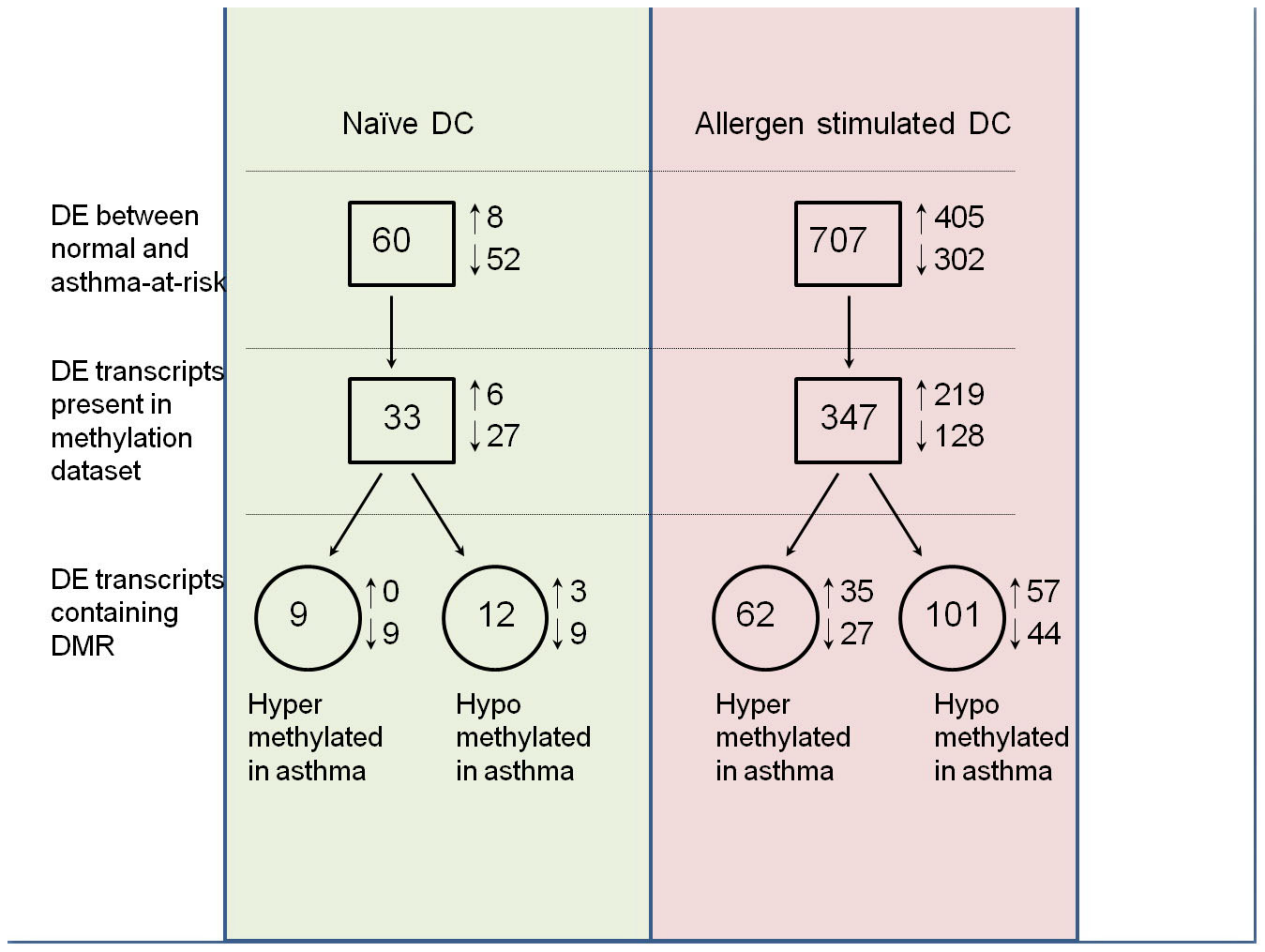
Number of transcripts differentially methylated and differentially expressed between asthma-at-risk DCs and controls.

To facilitate comparison between gene expression and DNA methylation data, we combined methylation and expression data for 9,964 transcripts which were represented on both platforms. Relevant numbers of differentially expressed (DE) transcripts which contain DMRs are schematically represented in Fig. 1.

Analysis of the Affymetrix expression data identified 60 differentially expressed (DE) transcripts in naïve DC (FDR 0.05 and fold change 1.25 or greater) out of 16,963 transcripts represented on the array. Methylation data was available for 33 of the 60 transcripts. We found that only 18 transcripts were both differentially expressed and differentially methylated in naïve DC (55% of transcripts for which both expression and methylation data are available). Three transcripts contained both hypo- and hyper-methylated DMRs. The majority (2/3) of the transcripts changed expression in the direction predicted by a corresponding shift in DNA methylation (following the predominant paradigm that lower methylation is associated with higher expression). The expression changes were minor in magnitude for these targets.

**Figure 2:**
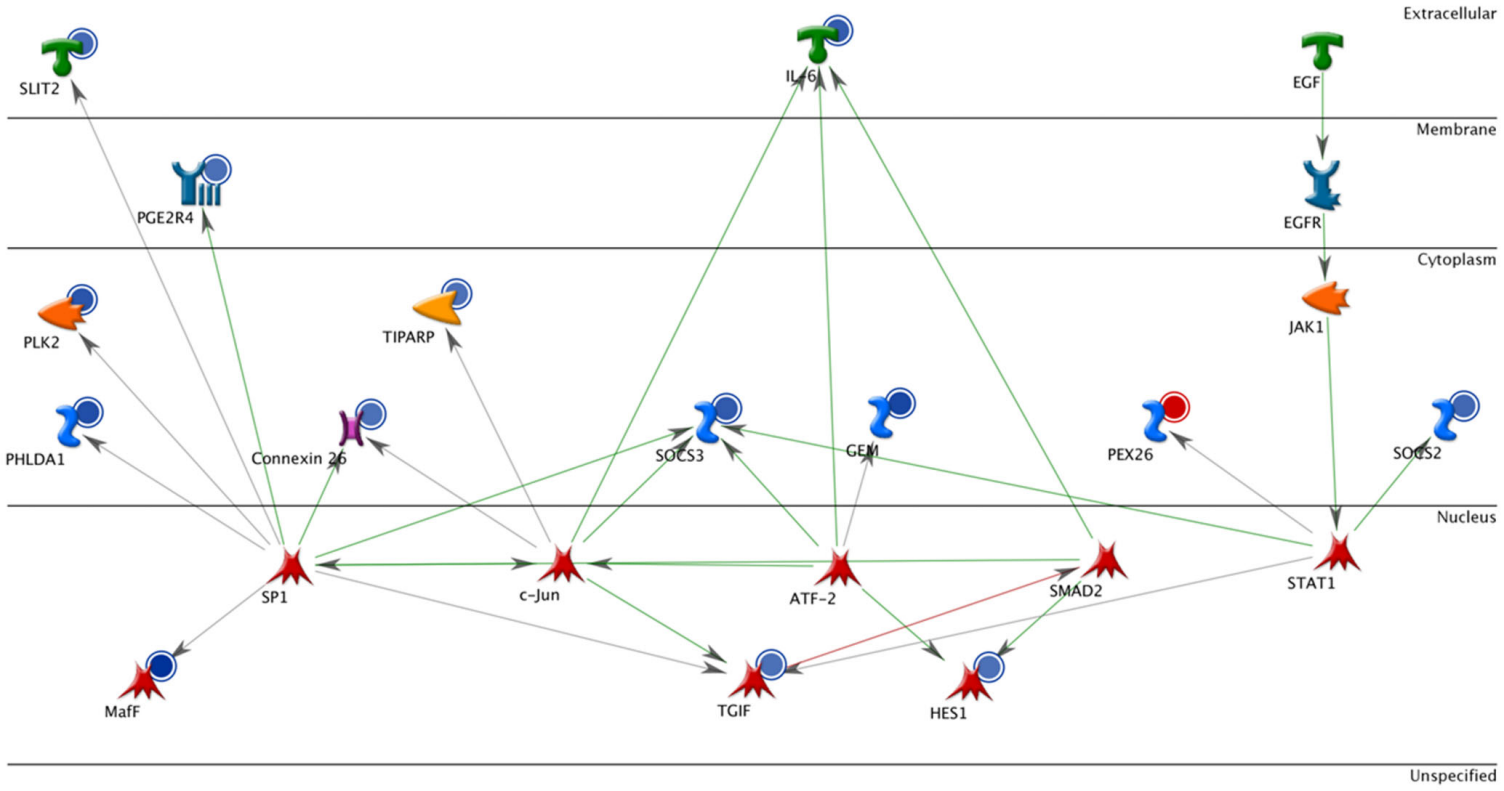
Transcripts showing both DNA methylation and expression changes between naïve normal and asthma-at-risk DCs form a small interaction network focused on regulation of a pleiotropic cytokine IL-6. Interacting factors are depicted in various shapes depending on their biological nature, connecting arrows indicate known links. Blue circles indicate transcripts down-regulated in asthma-at-risk DCs, red – up-regulated.

**Figure 3:**
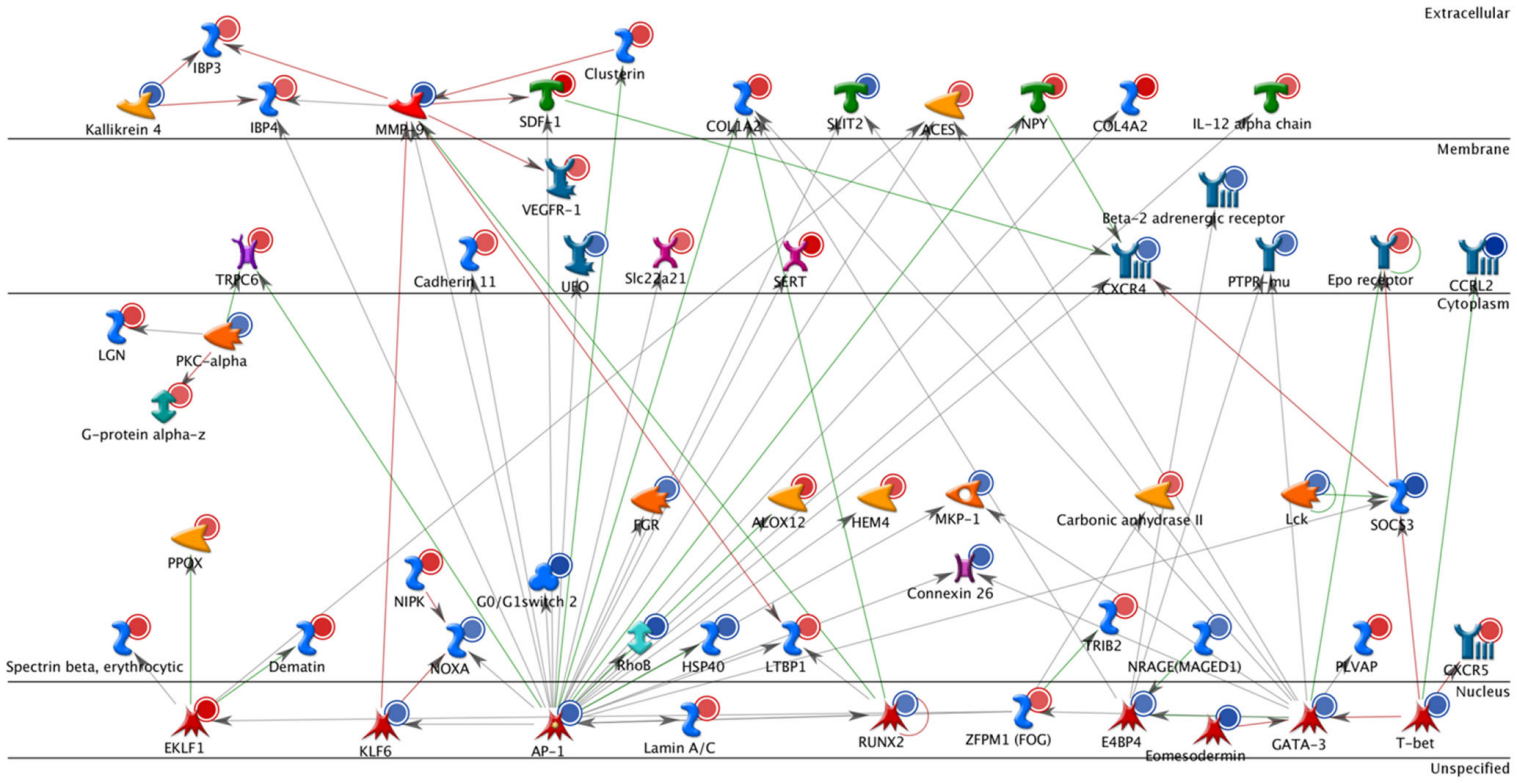
Interaction network of transcripts with significant change in DNA methylation at birth that show significant transcriptional change later in life upon the first encounter with allergen. Interacting factors are depicted in various shapes depending on their biological nature, connecting arrows indicate known links. Blue circles indicate transcripts down-regulated in asthma-at-risk DCs, red – up-regulated.

Pathway analysis of the 18-target list revealed three canonical pathways enriched with differentially-expressed and differentially-methylated genes in naïve DC: the Jak-Stat pathway, which is mostly down-regulated in asthma-at-risk DC (and remains significant even if only down-regulated genes are analyzed), the RAR-alpha pathway (Fig. 2), and a cluster of genes in the VEGF pathway. Remarkably, 15 out of 18 transcripts belong to the interaction network focused on the regulation of IL-6 (Fig. 2), a cytokine crucial in early asthma development [21]. This suggests that deregulation of the IL-6 methylation profile could play an important role in a pro-allergic skew of allergen-naïve DCs, and possibly is an early indication of asthma predisposition. The fact that the 18 genes belong to pathways relevant to asthma is encouraging and supports the biological significance of our analysis. It is noteworthy however, that majority of DE genes in these pathways are not differentially expressed in stimulated DC (compare Figs. 2 and 3) suggesting they may not play role in the allergen response past the sensitization.

Hence, analysis of allergen-naive samples identified multiple epigenetic changes induced by maternal allergy but these were not linked to major background transcription alterations on their own (with the exception of a minor transcriptional shift in the IL-6 signaling pathway). These findings can now be compared to what was found after allergen stimulation.

### Upon allergen sensitization robust transcriptional changes are seen among transcripts showing epigenetic alterations at birth

We have found a substantially greater number of differentially expressed transcripts after allergen stimulation. 707 transcripts were differentially expressed overall in asthma-at-risk vs naive DC (FDR 0.05 and fold change 1.25 or greater), and 347 of them were present in the methylation ‘hit list’ as differentially methylated in asthma-at-risk vs naive DC. 62 of the transcripts were associated with unmethylated DMRs in normal naïve DC, and 101 in asthma-at-risk (25 transcripts contain both). Similar to naïve DC, a group of transcripts contained both hyper- and hypo- methylated DMRs. We observed similar numbers of up- and down-regulated transcripts in the stimulated group, with slightly more up-regulation in hypo-methylated subgroup (Fig. 1).

About one-third of the DE and DM transcripts from allergen-stimulated DCs interact functionally with each other, some of them with multiple interactions (Fig. 3). Among them are a number of genes previously found to be involved in allergic responses, such as Runx2, MMP9, CXCR4 and 5, IL-12, beta-adrenoreceptor, cadherin, AP1, VEGF-R1, etc. While many pathways are affected by transcriptional changes in stimulated DC, the focal points of the changes appear to be transcription factors. The top 30 significant transcriptional networks include several subunits of NFkB, and several STAT genes, which are involved in regulation of immune response. However, other transcription networks, associated with more general functions such as cell proliferation and differentiation are also prominent.

Because each gene is targeted by several probes in the methylation array, we sought to determine whether there are targets that have both hyper- and hypomethylated areas, and we identified only 8 such genes for the naïve (Table S1) and 61 genes for allergen-sensitized mice (Table S2).

**Figure 4:**
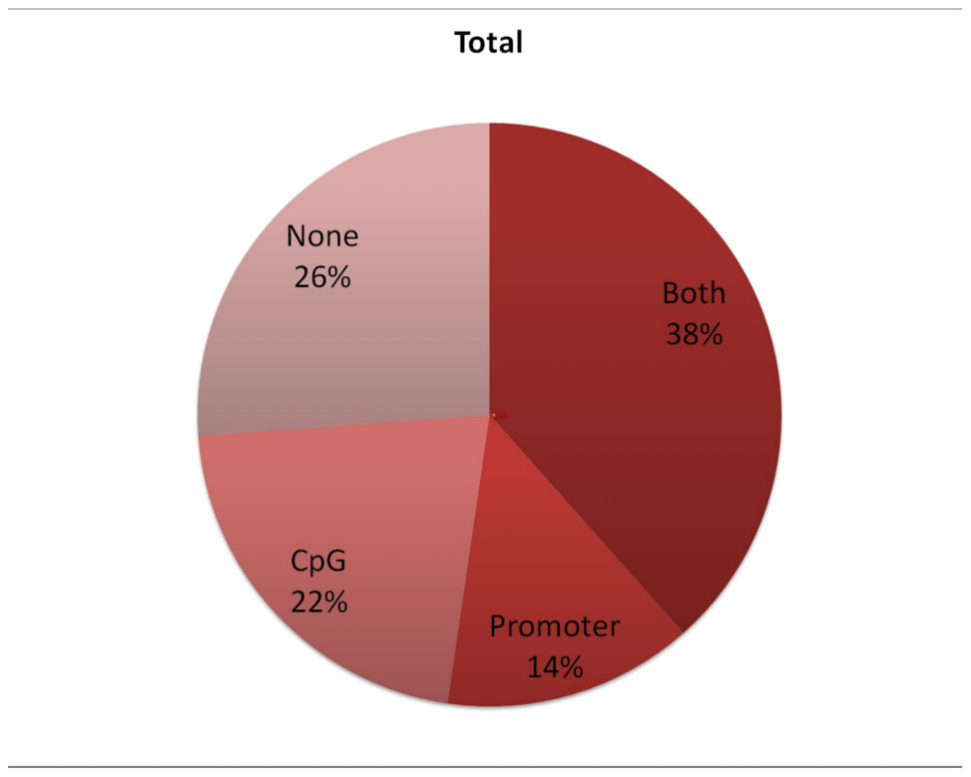
In the allergen stimulated group DCs most genes with significant change in transcription (DE) had altered methylation either in the promoter (14%), CpG island (22%) or both (38%) (of all overalpping DMR-DE). Only 26% of the genes had changes elsewhere in the body of the transcript.

For allergen-sensitized mice most of the methylation changes occurred either within promoters of genes or overlapped with a nearby CpG island; a small proportion of probesets (∼26%) were mapped to areas that are unlikely to include key transcription regulation elements (Fig. 4).

The presence of DE transcripts with multiple DMRs altered in opposing directions (hypo- and hyper-) presented an opportunity to analyze the role of relative position of CpG sites in altering transcription. 65 such transcripts were found for allergen-sensitized DC samples with FDR for DE 0.05 (no restriction on fold change). The DMRs associated with these transcripts were categorized as overlapping with promoter, CpG island, both or neither. A DMR was selected as “influential” if its methylation changed in accordance with subsequent change in transcription. If more than one “influential” DMR was present, one was picked with following priority: Promoter and CpG island>Promoter>CpG island>neither. While this approach is biased against CpG islands, only two transcripts were affected (having separate “influential” probes in promoter only and in CpG island only), both down-regulated.

Approximately ¾ of the transcripts have the potentially ‘influential’ probes within expected regulatory regions – promoters or CpG islands or both (72% of up-regulated transcripts, 76% of down-regulated, and 74% overall, Fig. 4). In more than 1/3 of the transcripts such probes are located in CpG islands within promoter region (43%, 35%, and 38% respectively). Interestingly, CpG islands outside of promoter regions (but within the body of a transcript) appear to influence transcription quite often, especially in case of down-regulated transcripts (30%, even with minor bias against such probes). Ultimately, epigenetic alterations at several regions simultaneously may be necessary for a subsequent transcriptional shift.

Genes that were downregulated were more likely to have aberrant methylation in the CpG islands than in the promoter (30% vs 11% in islands and 11% vs 18% in promoters). This finding supports the recent paradigm that methylation of the CpG islands has a stronger transcriptional significance than methylation of promoter sequences.

In summary, upon allergen sensitization the DCs of asthma-at-risk neonates show significant transcriptional change compared to controls; the majority of up- or downregulated genes have pre-existing methylation changes (at birth) indicating that maternal transmission of asthma risk associated with the dysfunction of DCs is largely an epigenetic phenomenon.

## Discussion

Recent publications demonstrate special importance of the epigenetic changes acquired during perinatal development to the future health. Maternal asthma transmits an increased asthma risk to neonates, both in humans and in our mouse model. Others show in a similar model that such risk may be transmitted transgenerationally, and postulate epigenetic mechanisms [22]–[24]. We have previously identified neonatal DC as a critical cell that conveys this asthma risk to normal recipients in adoptive transfer experiments [20].

This report demonstrates that neonates at-risk of asthma are born with substantial genome-wide DNA methylation changes in their DCs due to maternal allergy. We based our hypothesis on the paradigm that allergen sensitization in responsive subjects leads to genome-wide changes in transcription of immune cells as they activate. The premise of this study was that many of these changes are preceded (and potentially caused) by epigenetic alterations in key regulatory sequences that arise as a result of maternal influence.

As we predicted, transcriptional differences in the allergen-naïve neonates are minimal. While the number of statistically significant DE transcripts (n = 60) is not negligible, for the majority of these transcripts (80%) the expression difference between normal and asthma-at-risk DC does not exceed 1.5 fold, and only one transcript (Fkbp5) changes more than two-fold. One of the limitations of such small differences detected on a genome-wide scale is difficulty reproducing them in other models and other species. This relatively small magnitude of expression changes is in agreement with the absence of phenotypical difference between naïve normal and asthma-at-risk pups, including absence of any immunophenotypic differences of the surface of DCs itself that as we reported earlier. Among differentially expressed are the genes involved in the IL-6 pathway due to their participation in Jak-Stat and Rar-alpha signaling pathways; they seem to be the only early indicators of a pro-allergic skew.

However, upon allergen sensitization, strong transcriptional differences are seen in genes that also had methylation aberrations at birth. For the allergen-stimulated DCs, we found over 700 DE transcripts; more than 40 of them were over 2-fold different, and several showed as much as a 4-fold change. Juxtaposing the methylation and expression data shows that a large percentage (55 for naïve DCs and 40 for allergen-stimulated DCs) of DE transcripts come from genes with pre-existing methylation changes. It is also worth noting that for the allergen-sensitized DCs, transcriptional changes were not limited to genes with detectable methylation changes at birth. This is plausible in light of the prominent involvement of transcription factors in our observations suggesting that deregulation of transcriptional networks has its own effects on many targets that are not epigenetically altered, and previous data reporting constitutively unmethylated genes.

An interesting aspect is that approximately the same number of genes change in compliance with the biological paradigm that demethylation leads to increased expression (56), as the number that change ‘discordantly’ in the opposite direction (53), suggesting that either methylation is but one of the factors in the complex mechanisms of transcriptional regulation, or that methylation in some sites is more transcriptionally important than in adjacent sites. Thus, some of the DMRs may be located at non-regulatory sequences, and others may be unaffected by the putative signaling pathways activated in allergy response. On the other hand, not all DE genes harbor differentially methylated sites, implying that they are regulated by other means.

The direct interactions network in Fig. 3 identifies genes that are likely causative for the early-life asthma origin, based on this analysis and our prior work [20], [25]. Among these 57 genes which evidently interact with each other very well, there are 29 that we found have a known role in asthma or allergic inflammation. They are: Klf6 (Gene ID: 23849) [29], Lamin A/C (Gene ID: 16905) [30], E4BP4 (Gene ID: 18030) [31], Eomesodemin (Gene ID: 13813) [32], GATA-3 (Gene ID: 14462) [33], T-bet (Gene ID: 57765) [34], CXCR5 (Gene ID: 12145) [35], SOCS3(Gene ID: 12702) [36], Lck (Gene ID: 16818) and FGR(Gene ID: 14191) [37], MKP-1 (Gene ID: 19252) [38], ALOX12(Gene ID: 11684) [39], RhoB (Gene ID 11852) [40], G0/G1 switch 2(Gene ID: 14373) relevant to cell proliferation, NOXA (Gene ID: 58801) [41], Dematin (Gene ID: 13829) [42], PKC-alpha (Gene ID: 18750) [43], TRPC6 (Gene ID: 22068) [44], VEGFR-1(Gene ID: 14254) [45], CXCR4 (Gene ID: 12767) [46], Beta-2 adrenergic receptor(Gene ID: 11555) [47], Il12a (Gene ID: 16159) [48], NPY (Gene ID: 109648) [49], Slit2 (Gene ID: 20563) [50], Col1a2 (Gene ID: 12843) [51], Cxcl12 (Gene ID: 20315) [52], MMP-9 (Gene ID: 17395) [53], IBP3 (Gene ID: 16009) [54], Kallikrein 4 (Gene ID: 56640) [55]. The rest of the factors (genes) are less well characterized and comprise opportunities for future research.

It remains unclear what role is played by the remaining methylation aberrations not studied here, i.e. changes outside of the coding or regulatory areas and changes in the genes that do not transcriptionally respond. These DMR may be either a secondary effect of changes in cell functionality, or a ‘bystander’ para-phenomenon that plays no role, or may have unknown regulatory consequences that remain to be discovered. A few methodological limitations of our study should also be mentioned: first, mouse models of disease do not necessarily reproduce every detail of human disease; second, genome-wide studies carry inherent risks related to multiplicity of comparisons; third, information provided by pathway analysis methods relies on known interactions between factors, which means the scope may be limited by published knowledge.

Conclusion: Maternal asthma leads to epigenetic change in DCs of the neonate, and this is linked to their skewed state at birth towards producing allergy responses upon encounter with allergen. The methylation changes are diverse and complex: they include both hyper and hypomethylation; many are associated with changed expression, but some are not; conversely, some expression changes are and some are not directly linked to methylation status. This brings new light to the epigenetic origins of asthma, demonstrating that they are not as simple as we may have hoped. These data also highlight the need for methods for targeted manipulation of the epigenetic status of genes to allow precise testing of causal roles in functional effects.

## Methods

### Animals

BALB/C mice were obtained from Charles River Laboratories (Cambridge, MA). Animal care complied with the Guide for the Care and Use of Laboratory Animals and all experiments were approved by the Institutional Review Board/IACUC: Harvard Center for Comparative Medicine.

### Exposure protocol

The animal protocol uses is based on previous studies [14], [20], [25]–[27]. Briefly, maternal sensitization is achieved by initial i.p. injections of 5 µg OVA with 1 mg alum in 0.1 ml PBS into mice at 3 and 7 days of age. After weaning, female mice are exposed to aerosols of allergen (3% (w/v) OVA (grade V, Sigma-Aldrich) in PBS, pH 7.4) for 10 min on 3 consecutive days at 4, 8, and 12 wk of age, and then mated with normal male mice. The aerosol exposure is performed within individual compartments of a mouse pie chamber (Braintree Scientific, Braintree, MA) using a Pari IS2 nebulizer (Sun Medical Supply, Kansas City, KS) connected to air compressor (PulmoAID; DeVilbiss, Somerset, PA). These female mice were shown to consistently have strongly increased AHR and AI [25]–[27].

Typically to confirm the maternal transmission effect, offspring of these allergic and of control PBS-challenged mice are subjected to ‘suboptimal’ protocol. On day 4 after birth newborns receive a single i.p. injection of OVA with alum (an intentionally suboptimal dosage that normally does not result in significant AHR and AI in offspring of normal intact or PBS-challenged mothers). On days 13–15 of life, these baby mice are exposed to aerosolized OVA, as above. In this report only the ‘quality-assurance’ neonates were subjected to this testing. For genomic and epigenomic profiling used in this work the neonatal DCs were harvested at d. 14 from either allergen-naïve neonates (“Naive DC” in Figure 1), or from those that received a single sensitization injection of OVA+alum, but not the aerosols (“Allergen stimulated DC” in Figure 1).

### Cell purification

Splenic dendritic cells (DCs) were prepared from collagenase D -treated (Roche) sterile cell suspensions using positive selection (retaining of CD11c+ cells) via the MACS magnetic bead system (Miltenyi Biotec, Auburn, CA). Purity was routinely monitored via flow cytometry (FACSCanto II, Beckton-Dickinson) by labeling for CD11c and MHC-II. More than 95% of the purified cells were double positive for these antigens, and viability was >93% by propidium iodide or trypan blue staining. After purifications the cells were washed 2 times in LPS-free sterile PBS with 5% BSA and once in pure ice-cold LPS-free sterile PBS.

### Isolation of nucleic acids and PCR

DNA and RNA were isolated using Qiagen DNEasy and RNEasy kits, respectively, in complete adherence with the instructions. RNA isolations included the optional DNAse digestion step. Quality of the RNA was tested spectrophotometrically via Nanodrop and confirmed in real-time PCR for beta-actin prior to submission. For quality control and validation purposes (Figure S1) the real-time PCR protocol was based on the SYBR Green assay (Bio-Rad) running 95°C - 10′, then 35 cycles of 95°C - 20″; 55°C - 25″; 72°C - 20″, then standard melt curve.

### Epigenomic profiling

The DNA methylation assay was performed by Switchgear Genomics (Menlo Park, CA), and the data is publicly available in GEO (GSE13380). The dataset consists of 18 two-color chipsets (9 biological repeats from the offspring of normal mothers, and 9 from the offspring of asthmatic mothers). Each chipset contains 386,009 probes out of which 345,225 are experimental probes, and the rest are control probes, presumably not changing with treatment.

The DNA from each sample was divided in two equal parts, and one part was treated with methylation sensitive endonuclease. Subsequently, treated part of each sample was labeled with Cy3 dye, and untreated part with Cy5, and both were hybridized on the same chip.

### Epigenomic data analysis

Raw fluorescence intensity values provided by Switchgear Genomics were used in the analysis, performed with Bioconductor package limma [28]. Two chipsets (one of a normal sample, and one of asthma-at-risk) were excluded from the analysis on the basis of their small interquartile difference, likely due to endonuclease treatment failure. Remaining 16 samples were first normalized within arrays using the “control” method. This method fits a global loess curve through a set of control (non-changing) spots, and applies the curve to the rest of the spots. The quantile method was used for between-arrays normalization.

After filtering out control probes, linear modeling approach was used for differential methylation inference, with subsequent fitting of the contrasts for methylation in normal samples, methylation in asthma-at-risk samples, and difference in methylation between normal and asthma-at-risk samples. The null hypothesis for the first two contrasts was that a probe is methylated, and for the third contrast that there is no difference between normal and asthmatic samples. Finally, t statistics for each probe were moderated using Bayes empirical method, and corresponding p-values adjusted for multiple testing according to Benjamini-Hochberg. Probes with adjusted p-values 0.05 or less and log2 fold change of 0.5 or greater were selected as unmethylated in respective groups. 23 477 such probes were identified as demethylated only in normal samples, and 28 615 probes only in asthmatic samples. These probes were further filtered applying following conditions: 1) the log2 fold change for methylated group <0.5; 2) absolute difference in log2 fold change between groups >0.3. Resulting lists of probes were mapped to transcripts, promoter regions (−1000 to +500 bases from TSS), and CpG islands (UCSC Genome Browser, release mm8) with Bioconductor packages GenomicFeatures, GenomicRanges, and IRanges (www.bioconductor.org).

### Expression profiling

Gene expression was assayed on the Affymetrix mouse gene chip Mouse430A 2.0 (Affymetrix, Santa Clara, CA) at the Dana Farber Cancer Institute hybridization facility. Two datasets were used in the analysis. Set 1 consists of 4 groups: naïve normal, naïve asthma-at-risk, allergen stimulated normal and allergen stimulated asthma-at-risk; 4 biological replicates in each group. Set 2 consists of two groups – allergen stimulated normal and allergen stimulated asthma-at-risk, 5 biological replicates each, NuGEN amplified.

### Expression data analysis

One array from each of the sets was excluded as outlier based on principle component analysis. The datasets were preprocessed separately using quantile normalization and median polish summarization steps. We used alternative cell definition file (cdf) - “mouse430a2mmrefseq” – available from Brainarray microarray lab at the University of Michigan (http://brainarray.mbni.med.umich.edu/brainarray) for summarization. After preprocessing, differential expression inference was performed with Bioconductor package limma. Comparisons of interest were: naïve normal versus naïve asthma-at-risk, and stimulated normal versus stimulated asthmatic (offspring of asthmatic mothers). T statistics for each probeset were moderated using Bayes empirical method, and p-values adjusted for multiple testing according to Benjamini-Hochberg.

### Pathway analysis

Pathways analysis was performed with Metacore software (GeneGo Inc.). Annotated lists of genes of interest (DMRs overlapping to transcriptionally changed targets in naive DC, and DMRs overlapping to transcriptionally changed targets in allergen-stimulated DC) were uploaded to the portal and analyzed separately using the “direct interactions” algorithm. This analysis visualizes all known direct interactions of the genes in a list to each other, according to the proprietary Metacore database. We have used the default options, i.e. no filtering by tissue or disease.

## Supporting Information

Figure S1 **PCR validation of the microarray data.** We validated 5 semi-randomly selected targets from the microarray results, both up- and down-regulated and unchanged, via real-time PCR. Microarray data represent fold change of “asthma” vs “normal” expression in RMA values; PCR data represent the SYBR green assay results from the same RNA samples; Cq values for each target were normalized to 18S RNA and similar fold change of “asthma” vs “normal” expression was obtained. n = 5/group.
(TIF)

Table S1 **Naïve DCs.**
(DOC)

Table S2 **Allergen-sensitized DCs.**
(DOC)
