# Supplementary figures and images for: Link between Epigenomic Alterations and Genome-Wide Aberrant Transcriptional Response to Allergen in Dendritic Cells Conveying Maternal Asthma Risk
Source: PLoS One. 2013 Aug 12;8(8):e70387. doi: 10.1371/journal.pone.0070387 (PMC3741290; doi:10.1371/journal.pone.0070387)

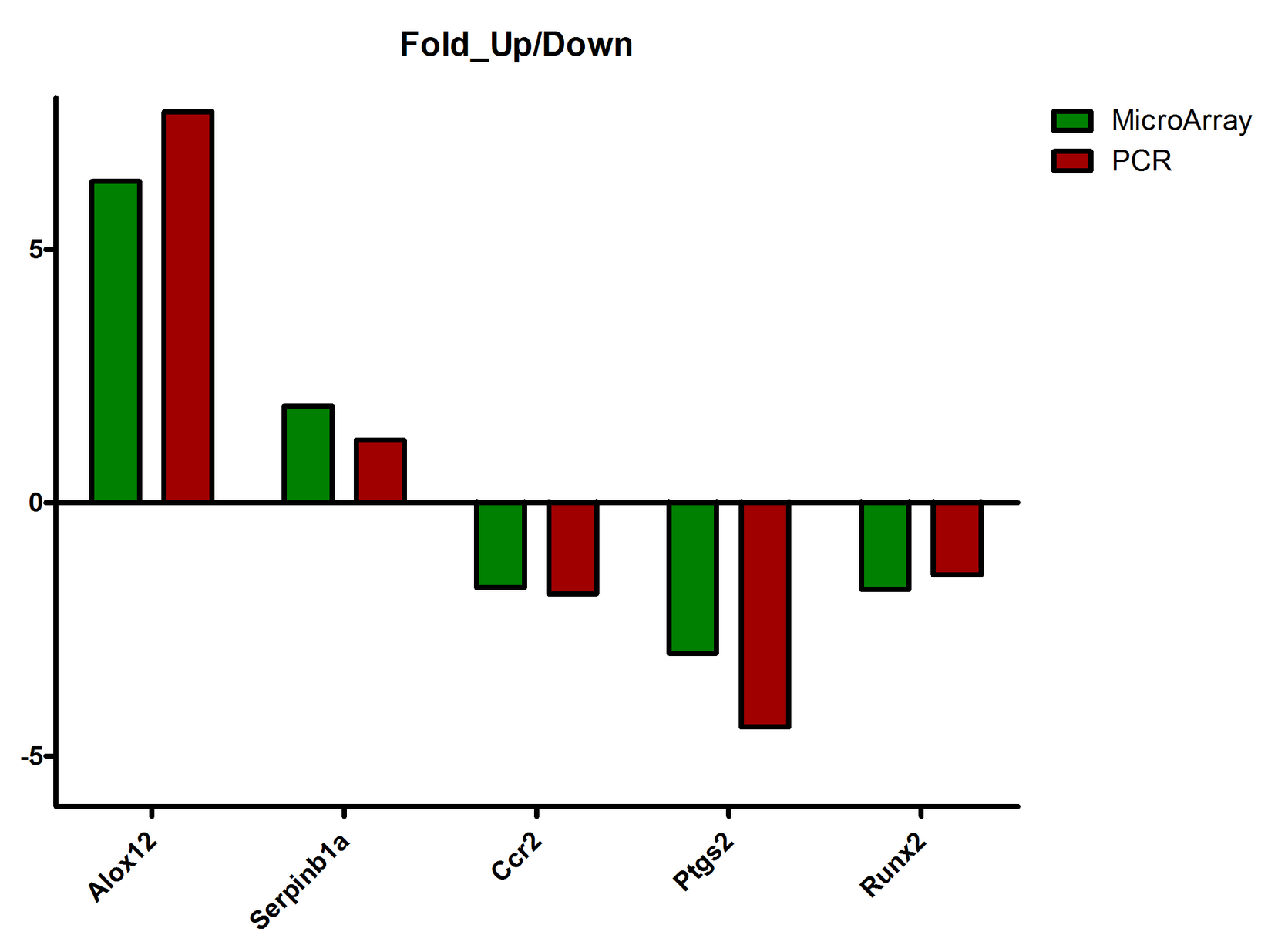

Supplement: Figure S1 — PCR validation of the microarray data. We validated 5 semi-randomly selected targets from the microarray results, both up- and down-regulated and unchanged, via real-time PCR. Microarray data represent fold change of “asthma” vs “normal” expression in RMA values; PCR data represent the SYBR green assay results from the same RNA samples; Cq values for each target were normalized to 18S RNA and similar fold change of “asthma” vs “normal” expression was obtained. n = 5/group. (TIF) [file pone.0070387.s001.tif]
