# Supplementary material for: Link between Epigenomic Alterations and Genome-Wide Aberrant Transcriptional Response to Allergen in Dendritic Cells Conveying Maternal Asthma Risk
Source: PLoS One. 2013 Aug 12;8(8):e70387. doi: 10.1371/journal.pone.0070387 (PMC3741290; doi:10.1371/journal.pone.0070387)
Supplement: Table S1 — Naïve DCs. (DOC) [file pone.0070387.s002.doc]

| Gene symbol | RefSeq | Probe ID | Direction of methylation change in asthma | Overlaps with promoter | Overlaps with CpG island |
| --- | --- | --- | --- | --- | --- |
| Hivep2 | NM_010437 | CHR10FS013655730 | hypo | yes | yes |
|  |  | CHR10FS013657530 | hypo | no | yes |
|  |  | CHR10FS013657670 | hypo | no | yes |
|  |  | CHR10FS013673383 | hyper | no | no |
| Socs3 | NM_007707 | CHR11FS117785200 | hyper | yes | yes |
|  |  | CHR11FS117785765 | hypo | yes | no |
|  |  | CHR11FS117785890 | hypo | yes | no |
| Hspa2 | NM_008301 | CHR12FS077322433 | hyper | yes | yes |
|  |  | CHR12FS077323413 | hypo | yes | yes |
|  |  | CHR12FS077323623 | hypo | yes | yes |
|  |  | CHR12FS077324573 | hypo | no | yes |
| Skil | NM_011386 | CHR03FS031285545 | hypo | yes | yes |
|  |  | CHR03FS031286160 | hyper | yes | yes |
| Skil | NM_001039090 | CHR03FS031285545 | hypo | yes | yes |
|  |  | CHR03FS031286160 | hyper | yes | yes |
| Pdgfra | NM_011058 | CHR05FS075434722 | hyper | no | yes |
|  |  | CHR05FS075435980 | hypo | no | no |
|  |  | CHR05FS075436182 | hypo | no | no |
|  |  | CHR05FS075438381 | hypo | no | yes |
|  |  | CHR05FS075444470 | hypo | no | no |
|  |  | CHR05FS075444595 | hypo | no | no |
| Gjb2 | NM_008125 | CHR14FS056058666 | hyper | yes | yes |
|  |  | CHR14FS056058806 | hypo | yes | yes |
|  |  | CHR14FS056059681 | hyper | yes | no |
| Ptger4 | NM_008965 | CHR15FS005189569 | hyper | no | yes |
|  |  | CHR15FS005191439 | hyper | yes | yes |
|  |  | CHR15FS005191726 | hypo | yes | no |
|  |  | CHR15FS005191989 | hypo | yes | no |

Table S1.

**Naïve DCs**
