# Supplementary material for: Link between Epigenomic Alterations and Genome-Wide Aberrant Transcriptional Response to Allergen in Dendritic Cells Conveying Maternal Asthma Risk
Source: PLoS One. 2013 Aug 12;8(8):e70387. doi: 10.1371/journal.pone.0070387 (PMC3741290; doi:10.1371/journal.pone.0070387)
Supplement: Table S2 — Allergen-sensitized DCs. (DOC) [file pone.0070387.s003.doc]

**Table S2.** Allergen-sensitized DCs

| Gene symbol | RefSeq | Probe ID | Direction of methylation change in asthma | Direction of expression change in asthma | Overlaps with promoter | Overlaps with CpG island |
| --- | --- | --- | --- | --- | --- | --- |
| P4ha1 | NM_011030 | CHR10FS058718612 | hyper | down | yes | yes |
|  |  | CHR10FS058718968 | hypo |  | yes | yes |
| Reep6 | NM_139292 | CHR10FS079732999 | hypo | up | yes | no |
|  |  | CHR10FS079733084 | hypo |  | yes | no |
|  |  | CHR10FS079733209 | hyper |  | yes | no |
| Gnptab | NM_001004164 | CHR10FS087808802 | hypo | down | yes | no |
|  |  | CHR10FS087809297 | hypo |  | yes | yes |
|  |  | CHR10FS087809655 | hypo |  | yes | no |
|  |  | CHR10FS087869993 | hyper |  | no | no |
| Tex2 | NM_198292 | CHR11FS106348321 | hypo | down | no | no |
|  |  | CHR11FS106428638 | hypo |  | yes | yes |
|  |  | CHR11FS106428778 | hyper |  | yes | yes |
| Prkca | NM_011101 | CHR11FS108135599 | hyper | down | no | no |
|  |  | CHR11FS108159120 | hyper |  | no | yes |
|  |  | CHR11FS108159400 | hypo |  | no | yes |
| Axin2 | NM_015732 | CHR11FS108736333 | hyper | down | yes | yes |
|  |  | CHR11FS108737385 | hyper |  | no | yes |
|  |  | CHR11FS108739658 | hypo |  | no | no |
| Socs3 | NM_007707 | CHR11FS117785200 | hyper | down | yes | yes |
|  |  | CHR11FS117785765 | hypo |  | yes | no |
|  |  | CHR11FS117785890 | hypo |  | yes | no |
| Slc16a3 | NM_030696 | CHR11FS120765565 | hyper | down | no | yes |
|  |  | CHR11FS120766995 | hypo |  | no | yes |
|  |  | CHR11FS120767285 | hyper |  | no | no |
| Gas7 | NM_008088 | CHR11FS067271464 | hypo | down | no | yes |
|  |  | CHR11FS067271759 | hyper |  | no | yes |
|  |  | CHR11FS067272180 | hyper |  | no | no |
| Cacna1g | NM_009783 | CHR11FS094225504 | hyper | up | no | no |
|  |  | CHR11FS094289321 | hypo |  | yes | no |
|  |  | CHR11FS094290093 | hyper |  | yes | yes |
|  |  | CHR11FS094290294 | hyper |  | yes | yes |
|  |  | CHR11FS094290514 | hypo |  | yes | yes |
| Kcnk13 | NM_146037 | CHR12FS100365943 | hypo | down | yes | yes |
|  |  | CHR12FS100366945 | hyper |  | yes | yes |
|  |  | CHR12FS100373818 | hypo |  | no | no |
| Lgmn | NM_011175 | CHR12FS102814638 | hyper | down | no | no |
|  |  | CHR12FS102841074 | hypo |  | yes | no |
| Rhoj | NM_023275 | CHR12FS076293219 | hyper | up | no | no |
|  |  | CHR12FS076310504 | hyper |  | no | no |
|  |  | CHR12FS076310574 | hypo |  | no | no |
| Lrrc16a | NM_026825 | CHR13FS024043701 | hyper | down | no | no |
|  |  | CHR13FS024074988 | hyper |  | no | no |
|  |  | CHR13FS024287149 | hypo |  | no | no |
| Clybl | NM_029556 | CHR14FS121364607 | hypo | up | no | no |
|  |  | CHR14FS121392589 | hyper |  | no | no |
| Zfp503 | NM_145459 | CHR14FS020775015 | hyper | up | no | yes |
|  |  | CHR14FS020777085 | hypo |  | no | no |
|  |  | CHR14FS020777670 | hypo |  | yes | yes |
|  |  | CHR14FS020777950 | hypo |  | yes | yes |
| Gjb2 | NM_008125 | CHR14FS056058806 | hypo | down | yes | yes |
|  |  | CHR14FS056059681 | hyper |  | yes | no |
| Myo10 | NM_019472 | CHR15FS025703631 | hypo | down | no | no |
|  |  | CHR15FS025750439 | hyper |  | no | no |
| Ankrd33b | NM_026153 | CHR15FS031311045 | hypo | down | no | no |
|  |  | CHR15FS031311120 | hypo |  | no | no |
|  |  | CHR15FS031311893 | hyper |  | yes | yes |
| Ptger4 | NM_008965 | CHR15FS005189569 | hyper | down | no | yes |
|  |  | CHR15FS005191439 | hypo |  | yes | yes |
|  |  | CHR15FS005191726 | hypo |  | yes | no |
| Mtss1 | NM_144800 | CHR15FS058806668 | hypo | up | no | no |
|  |  | CHR15FS058899549 | hypo |  | no | no |
|  |  | CHR15FS058911574 | hypo |  | yes | yes |
|  |  | CHR15FS058912136 | hyper |  | yes | yes |
| Khdrbs3 | NM_010158 | CHR15FS068758043 | hyper | up | yes | no |
|  |  | CHR15FS068758113 | hypo |  | yes | no |
|  |  | CHR15FS068758313 | hyper |  | yes | no |
| Pdgfb | NM_011057 | CHR15FS079839837 | hyper | down | no | yes |
|  |  | CHR15FS079840607 | hypo |  | no | yes |
|  |  | CHR15FS079841643 | hypo |  | yes | yes |
| Shank3 | NM_021423 | CHR15FS089328252 | hyper | up | yes | no |
|  |  | CHR15FS089351351 | hypo |  | no | no |
|  |  | CHR15FS089351635 | hyper |  | no | no |
|  |  | CHR15FS089351977 | hyper |  | no | yes |
|  |  | CHR15FS089385718 | hypo |  | no | yes |
| Nfkbiz | NM_030612 | CHR16FS055746975 | hypo | down | no | yes |
|  |  | CHR16FS055763681 | hyper |  | yes | no |
| Abat | NM_172961 | CHR16FS008428673 | hyper | up | yes | yes |
|  |  | CHR16FS008428803 | hypo |  | yes | yes |
| Notch3 | NM_008716 | CHR17FS031894602 | hyper | down | no | yes |
|  |  | CHR17FS031894867 | hypo |  | no | yes |
|  |  | CHR17FS031895544 | hyper |  | yes | no |
|  |  | CHR17FS031895599 | hypo |  | yes | no |
| Gtpbp2 | NM_019581 | CHR17FS045624687 | hyper | up | yes | no |
|  |  | CHR17FS045625113 | hypo |  | yes | no |
| Epb4.1l3 | NM_013813 | CHR17FS069063113 | hypo | down | no | no |
|  |  | CHR17FS069101230 | hypo |  | no | no |
|  |  | CHR17FS069193049 | hyper |  | no | no |
| Celf4 | NM_133195 | CHR18FS025647976 | hypo | down | no | no |
|  |  | CHR18FS025648131 | hyper |  | no | no |
|  |  | CHR18FS025676518 | hyper |  | no | no |
|  |  | CHR18FS025733232 | hyper |  | no | no |
|  |  | CHR18FS025897827 | hyper |  | yes | yes |
| Tlx1 | NM_021901 | CHR19FS045204408 | hyper | up | yes | yes |
|  |  | CHR19FS045209210 | hyper |  | no | yes |
|  |  | CHR19FS045209435 | hypo |  | no | yes |
| Cacybp | NM_009786 | CHR01FS162048979 | hypo | down | yes | no |
|  |  | CHR01FS162049269 | hyper |  | yes | yes |
|  |  | CHR01FS162049399 | hyper |  | yes | yes |
| Efhd1 | NM_028889 | CHR01FS089095670 | hyper | up | yes | yes |
|  |  | CHR01FS089096180 | hyper |  | no | no |
|  |  | CHR01FS089096510 | hypo |  | no | no |
| Blcap | NM_016916 | CHR02FS157252836 | hyper | down | no | no |
|  |  | CHR02FS157258285 | hypo |  | yes | no |
| Pkp4 | NM_026361 | CHR02FS058961448 | hypo | down | yes | no |
|  |  | CHR02FS058962903 | hyper |  | no | yes |
| Tanc1 | NM_198294 | CHR02FS059412998 | hyper | up | yes | yes |
|  |  | CHR02FS059413083 | hyper |  | yes | yes |
|  |  | CHR02FS059413425 | hyper |  | no | yes |
|  |  | CHR02FS059519807 | hyper |  | no | no |
|  |  | CHR02FS059631253 | hypo |  | no | no |
| Stk39 | NM_016866 | CHR02FS068229174 | hypo | down | no | no |
|  |  | CHR02FS068237066 | hypo |  | no | no |
|  |  | CHR02FS068272022 | hyper |  | no | yes |
| Sort1 | NM_019972 | CHR03FS108411654 | hypo | down | yes | no |
|  |  | CHR03FS108412640 | hyper |  | yes | no |
|  |  | CHR03FS108412778 | hypo |  | no | no |
|  |  | CHR03FS108416544 | hypo |  | no | no |
| Car2 | NM_009801 | CHR03FS014862884 | hyper | up | yes | yes |
|  |  | CHR03FS014863449 | hypo |  | yes | yes |
|  |  | CHR03FS014863659 | hypo |  | yes | yes |
| Foxo1 | NM_019739 | CHR03FS052356119 | hypo | up | yes | yes |
|  |  | CHR03FS052356539 | hypo |  | yes | yes |
|  |  | CHR03FS052356684 | hyper |  | yes | yes |
| Fhdc1 | NM_001033301 | CHR03FS084566525 | hyper | up | yes | yes |
|  |  | CHR03FS084566745 | hypo |  | yes | yes |
| Jun | NM_010591 | CHR04FS094543273 | hyper | down | no | yes |
|  |  | CHR04FS094544940 | hypo |  | yes | yes |
| Hip1 | NM_146001 | CHR05FS135704632 | hyper | down | no | no |
|  |  | CHR05FS135774247 | hypo |  | no | no |
|  |  | CHR05FS135829928 | hypo |  | yes | no |
| Flt1 | NM_010228 | CHR05FS147976304 | hypo | up | no | no |
|  |  | CHR05FS147991985 | hyper |  | no | no |
|  |  | CHR05FS148036776 | hypo |  | yes | yes |
| En2 | NM_010134 | CHR05FS028496637 | hyper | up | yes | yes |
|  |  | CHR05FS028496717 | hyper |  | yes | yes |
|  |  | CHR05FS028497837 | hyper |  | no | yes |
|  |  | CHR05FS028498530 | hypo |  | no | no |
|  |  | CHR05FS028498612 | hypo |  | no | no |
|  |  | CHR05FS028499547 | hypo |  | no | no |
|  |  | CHR05FS028499847 | hypo |  | no | no |
|  |  | CHR05FS028501254 | hypo |  | no | no |
|  |  | CHR05FS028501999 | hyper |  | no | no |
| Lap3 | NM_024434 | CHR05FS045781785 | hyper | down | yes | yes |
|  |  | CHR05FS045781915 | hypo |  | yes | yes |
|  |  | CHR05FS045782125 | hypo |  | yes | no |
| Rell1 | NM_145923 | CHR05FS064204010 | hypo | down | no | no |
|  |  | CHR05FS064204227 | hypo |  | no | no |
|  |  | CHR05FS064247525 | hyper |  | yes | yes |
|  |  | CHR05FS064247800 | hyper |  | yes | yes |
|  |  | CHR05FS064248291 | hypo |  | yes | no |
| Abcb1b | NM_011075 | CHR05FS008803958 | hypo | down | yes | no |
|  |  | CHR05FS008804168 | hyper |  | yes | no |
|  |  | CHR05FS008804533 | hyper |  | yes | yes |
| Podxl | NM_013723 | CHR06FS031493489 | hypo | up | no | no |
|  |  | CHR06FS031493559 | hyper |  | yes | yes |
| Gata2 | NM_008090 | CHR06FS088164950 | hypo | up | no | yes |
|  |  | CHR06FS088166053 | hyper |  | no | no |
| Zxdc | NM_173002 | CHR06FS090334910 | hypo | up | yes | yes |
|  |  | CHR06FS090335195 | hyper |  | yes | yes |
| Prkcdbp | NM_028444 | CHR07FS105355517 | hyper | up | yes | no |
|  |  | CHR07FS105356077 | hypo |  | yes | yes |
|  |  | CHR07FS105356157 | hypo |  | yes | yes |
| Kif22 | NM_145588 | CHR07FS126824407 | hypo | up | no | no |
|  |  | CHR07FS126833238 | hyper |  | yes | no |
|  |  | CHR07FS126833658 | hypo |  | yes | yes |
| Qprt | NM_133686 | CHR07FS126907827 | hypo | down | no | no |
|  |  | CHR07FS126907902 | hyper |  | no | no |
| Uros | NM_009479 | CHR07FS133547059 | hypo | up | no | yes |
|  |  | CHR07FS133547134 | hypo |  | yes | yes |
|  |  | CHR07FS133548044 | hyper |  | yes | no |
| Tspan4 | NM_053082 | CHR07FS141333006 | hypo | down | no | no |
|  |  | CHR07FS141333488 | hyper |  | no | yes |
|  |  | CHR07FS141333923 | hypo |  | no | no |
| Fosb | NM_008036 | CHR07FS018467214 | hyper | down | no | yes |
|  |  | CHR07FS018468670 | hypo |  | yes | yes |
| Zfp112 | NM_021307 | CHR07FS023820911 | hypo | up | yes | no |
|  |  | CHR07FS023821131 | hyper |  | yes | no |
| Odz4 | NM_011858 | CHR07FS096087525 | hypo | down | no | no |
|  |  | CHR07FS096261016 | hypo |  | no | no |
|  |  | CHR07FS096323135 | hypo |  | no | no |
|  |  | CHR07FS096764135 | hyper |  | no | no |
| Aars | NM_146217 | CHR08FS113920445 | hypo | up | yes | no |
|  |  | CHR08FS113943271 | hyper |  | no | yes |
| Csgalnact1 | NM_172753 | CHR08FS071552982 | hypo | up | no | no |
|  |  | CHR08FS071663907 | hyper |  | yes | yes |
| Celsr3 | NM_080437 | CHR09FS108683531 | hyper | down | yes | no |
|  |  | CHR09FS108683591 | hypo |  | yes | no |
|  |  | CHR09FS108684201 | hypo |  | no | no |
|  |  | CHR09FS108689712 | hyper |  | no | yes |
| Ccr9 | NM_009913 | CHR09FS123527565 | hyper | down | yes | yes |
|  |  | CHR09FS123527565 | hyper |  | no | yes |
|  |  | CHR09FS123527650 | hypo |  | no | yes |
| Smad6 | NM_008542 | CHR09FS063751623 | hyper | down | no | yes |
|  |  | CHR09FS063818354 | hyper |  | no | yes |
|  |  | CHR09FS063819335 | hypo |  | yes | yes |
| Rbpms2 | NM_028030 | CHR09FS065428478 | hyper | up | yes | yes |
|  |  | CHR09FS065429039 | hyper |  | yes | yes |
|  |  | CHR09FS065429178 | hyper |  | no | yes |
|  |  | CHR09FS065429748 | hypo |  | no | no |
